# Racial/ethnic differences in Life’s Essential 8 and polysocial risk as an effect modifier among women of reproductive age

**DOI:** 10.1016/j.ajpc.2026.101743

**Published:** 2026-07-11

**Authors:** Feodora Etelier, Ateh Stanislas Ketum, Asma Rayani, Ana Baez Mateo, Lilian Hernandez, Kwabena Nana Achiaw Dankwa, Kwabena Odei-Kumi, Chioma Ezuma, Dhananjay Vaidya, Pamela Ouyang, Melissa D. Hladek, Garima Sharma, Yvonne Commodore-Mensah, Faith E. Metlock

**Affiliations:** a Johns Hopkins University Krieger School of Arts and Sciences, Baltimore, MD, USA; b Johns Hopkins School of Nursing, Baltimore, MD, USA; c Johns Hopkins Bloomberg School of Public Health, Baltimore, MD, USA; d Johns Hopkins School of Medicine, Baltimore, MD, USA; e Inova Heart and Vascular Institute, Inova Fairfax Medical Campus, Fairfax, VA, USA

**Keywords:** Life's essential 8, Cardiovascular health, Cardiovascular risk, Racial disparities in women, Hypertension, And reproductive age

## 1 Introduction

Racially/ethnically minoritized women of reproductive age face a disproportionate burden of cardiovascular risk factors shaped by social determinants of health (SDoH) [1,2]. The American Heart Association’s Life’s Essential 8 (LE8) framework was developed to assess cardiovascular health (CVH) across key modifiable behaviors and factors: diet, physical activity, nicotine exposure, sleep, body mass index, blood lipids, blood glucose, and blood pressure [3]. Although national LE8 studies show suboptimal CVH and persistent racial/ethnic differences among U.S. adults, less is known about these patterns among women of reproductive age [4]. Limited research has examined whether polysocial risk, a cumulative measure of social disadvantage, modifies associations between race/ethnicity, and CVH [5]. This study examined racial/ethnic differences in individual LE8 indicators among Social Determinants of Hypertension in Women of Reproductive Age (SAFE HEART) study [6] participants and explored whether polysocial risk modified associations between race/ethnicity, and LE8 outcomes.

## 2 Methods

### 2.1 Study design/population

The SAFE HEART study [6] has been described elsewhere. Briefly, the SAFE HEART study is a community-engaged study on CVH, CVH literacy, and SDoH among women of reproductive age. This cross-sectional analysis included women aged 18 to 50 years recruited through the American Heart Association Research Goes Red Registry and community-based outreach in the Baltimore–Washington, D.C., metropolitan area who completed study procedures in English or Spanish.

### 2.2 Race/ethnicity ascertainment

Participants were categorized as non-Hispanic (NH) White, NH Black, Hispanic/Other. The Hispanic/Other category included Hispanic participants, NH Asian participants, and participants who identified as NH Other, which were combined due to small subgroup sample sizes. NH White women served as the reference group in all regression analyses.

### 2.3 Ascertainment of Life’s Essential 8 outcomes

Participants were asked whether they had ever been told by a doctor or other health professional that they had hypertension, high cholesterol, or diabetes, and responses were categorized as yes or no. Body mass index (BMI) was calculated from measured height and weight collected during the study, with high BMI defined as >25 kg/m². Smoking status was assessed using the question, “Have you smoked at least 100 cigarettes in your entire life?” and dichotomized as yes or no. Physical activity was assessed by self-report and classified as low if participants reported fewer than 150 min of moderate physical activity or exercise per week, excluding occupational activity. Diet was assessed using self-reported fruit and vegetable intake and categorized as low if participants reported consuming fewer than 5 servings per day. Sleep duration was classified as poor if participants reported sleeping fewer than 7 h or >9 h per night.

### 2.4 Ascertainment of polysocial risk score

Polysocial risk was measured using a 14-item score across 7 domains: sociodemographic characteristics, living situation, food security, transportation, utilities, safety, and financial strain. Each adverse factor was coded as 1 and summed, with total scores categorized into tertiles representing low, moderate, and high polysocial risk.

### 2.5 Statistical analysis

To evaluate racial/ethnic differences in CVH factors, we fit separate multivariable logistic regression models for each LE8 outcome, adjusting for age and polysocial risk. NH White women were used as the reference group for all models. We assessed for interaction between race/ethnicity and polysocial risk tertile across all eight LE8 components. Significant effect modification was observed for hyperlipidemia (p = 0.025) and low fruit/vegetable intake (p = 0.045); stratified results for these outcomes are presented in Fig. 1, Panel B. For the remaining six components, the interaction was not significant (all p > 0.05), and pooled adjusted odds ratios and 95% confidence intervals are reported in the results section. Statistical significance was defined as a two-sided p-value <0.05. All analyses were conducted using Stata version 18 (StataCorp, College Station, TX, USA). All participants provided informed consent, and the study protocol was approved by the Johns Hopkins University Institutional Review Board.

**Fig. 1:**
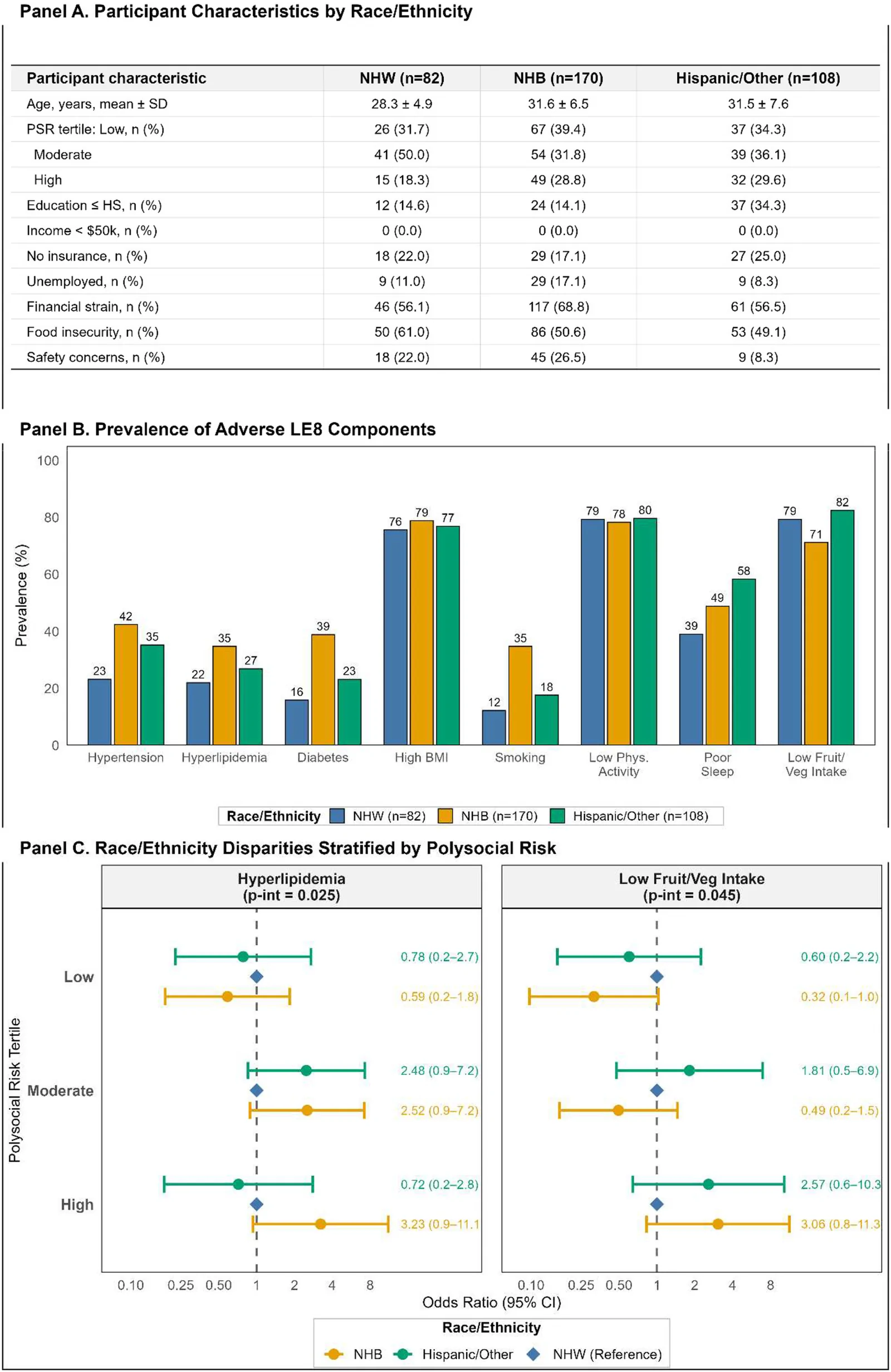
Participant characteristics, adverse Life’s Essential 8 components, and racial/ethnic differences stratified by polysocial risk. Panel A presents participant characteristics by race/ethnicity among women enrolled in the SAFE HEART Study. Panel B shows the prevalence of adverse Life’s Essential 8 (LE8) components across non-Hispanic White (NHW), non-Hispanic Black (NHB), and Hispanic/Other participants. Panel C displays adjusted odds ratios and 95% confidence intervals for racial/ethnic differences in hyperlipidemia and low fruit/vegetable intake stratified by polysocial risk tertile; NHW participants served as the reference group. Models were adjusted for age and polysocial risk. P-int indicates the p-value for the race/ethnicity × polysocial risk interaction test for each respective outcome.

## 3 Results

### 3.1 Sociodemographic and clinical characteristics

A total of 360 (mean age: 30.9 ± 6.7 years) participants were enrolled in the SAFE HEART study, including 82 NH White, 170 NH Black, and 108 Hispanic/Other. Several characteristics differed by race/ethnicity. Hispanic/Other women were more likely to report a high school education or less, while NH Black and Hispanic/Other women were more likely to report not owning their home. Housing condition concerns were highest among Hispanic/Other and NH White women, utility difficulties were more common among NH White and NH Black women, and neighborhood safety concerns were highest among NH Black women (**Figure, Panel A**). Adverse LE8 components were common across racial/ethnic groups, particularly high BMI, low physical activity, and low fruit/vegetable intake. NH Black women had the highest prevalence of hypertension, hyperlipidemia, diabetes, and smoking, while Hispanic/Other women had the highest prevalence of poor sleep and low fruit/vegetable intake (**Figure, Panel B**).

### 3.2 Racial/ethnic differences in Life’s Essential 8 outcomes

Among outcomes without significant effect modification, NH Black women had significantly higher odds of hypertension (OR 2.21; 95% CI 1.16 - 4.20), diabetes (OR 3.23; 95% CI 1.60 - 6.53), and current smoking (OR 3.47; 95% CI 1.60 - 7.52) compared to NH White women after adjusting for polysocial risk tertile and age. Hispanic/Other women had significantly higher odds of poor sleep (OR 2.12; 95% CI 1.16 −3.88). No significant racial/ethnic differences were observed for high BMI among NH Black or Hispanic/Other women compared with NH White women (OR 1.09, 95% CI 0.56–2.11; OR 0.92, 95% CI 0.45–1.88, respectively), or for low physical activity (OR 0.96, 95% CI 0.49–1.87; OR 1.04, 95% CI 0.50–2.16, respectively).

For the two outcomes with significant effect modification, the pattern of racial/ethnic differences varied across polysocial risk strata. For hyperlipidemia, NH Black and Hispanic/Other women had similar or lower odds compared with NH White women at low polysocial risk, while elevated odds emerged at moderate polysocial risk. At high polysocial risk, the pattern diverged, with elevated odds persisting among NH Black women but not Hispanic/Other women. A similar gradient was observed for low fruit/vegetable intake, where NH Black and Hispanic/Other women shifted from lower odds at low polysocial risk to higher odds at high polysocial risk. (**Figure, Panel C**).

## 4 Discussion

Among this community-engaged sample of women of reproductive age, polysocial risk modified racial/ethnic differences in select LE8 outcomes, specifically hyperlipidemia and low fruit/vegetable intake. Although stratum-specific estimates were limited by small sample size, significant interactions indicated that racial/ethnic differences in hyperlipidemia and low fruit/vegetable intake varied across polysocial risk levels. These findings highlight that racial/ethnic differences in CVH are shaped by social context and may become more pronounced under conditions of greater cumulative social disadvantage. While NH Black women also had higher odds of hypertension, diabetes, and current smoking, and Hispanic/Other women had higher odds of poor sleep, the effect modification findings highlight polysocial risk as an important context for understanding how LE8 disparities emerge during the reproductive years.

By focusing on the full age spectrum of women of reproductive age recruited from diverse community-based settings, our findings extend national LE8 research [4,7] and suggest that cumulative social disadvantage may shape racial/ethnic differences in specific LE8 outcomes, particularly diet-related behaviors and cardiometabolic risk. These results support pairing LE8 assessment with social risk screening in community-based, preconception, and postpartum settings [5]. Strengths include the focus on women of reproductive age and community-engaged recruitment. Limitations include the cross-sectional design, self-reported clinician diagnoses for hypertension, hyperlipidemia, and diabetes, and potential residual confounding. Future studies should include larger samples and more detailed measures of structural racism, discrimination, neighborhood context, healthcare access, and chronic stress.

## 5 Conclusion

Cumulative social disadvantage may play an important role in shaping racial/ethnic differences in LE8 outcomes among women of reproductive age. Cardiovascular prevention strategies should integrate LE8 assessment with social risk screening to better identify and address inequities in CVH earlier in the life course.

## Sources of funding

This study was funded by the American Heart Association's Research Goes Red Grants 979462 and 147554. FEM is supported by the National Institute of Nursing Research of the 10.13039/100000002National Institutes of Health under award Number T32NR020315 and the 10.13039/100000968American Heart Association under award number 24PRE12438460. YCM is supported by the American Heart Association Health Equity Research Network on the Prevention of Hypertension (Grant number: 882415) and 10.13039/100006545National Institute on Minority Health and Health Disparities (Grant number: P50MD017348-818). GS is supported by the American Heart Association under award number 979462.

## Disclosures

The authors have no disclosures to report.

## CRediT authorship contribution statement

**Feodora Etelier:** Writing – review & editing, Writing – original draft, Visualization, Validation, Investigation, Conceptualization. **Ateh Stanislas Ketum:** Writing – review & editing, Formal analysis, Data curation. **Asma Rayani:** Writing – review & editing. **Ana Baez Mateo:** Writing – review & editing. **Lilian Hernandez:** Writing – review & editing. **Kwabena Nana Achiaw Dankwa:** Writing – review & editing. **Kwabena Odei-Kumi:** Writing – review & editing. **Chioma Ezuma:** Writing – review & editing. **Dhananjay Vaidya:** Writing – review & editing. **Pamela Ouyang:** Writing – review & editing. **Melissa D. Hladek:** Writing – review & editing. **Garima Sharma:** Writing – review & editing, Resources, Methodology, Funding acquisition. **Yvonne Commodore-Mensah:** Writing – review & editing, Validation, Supervision, Resources, Project administration, Methodology, Investigation, Funding acquisition, Formal analysis, Data curation, Conceptualization. **Faith E. Metlock:** Writing – review & editing, Writing – original draft, Visualization, Validation, Supervision, Software, Resources, Project administration, Methodology, Investigation, Funding acquisition, Formal analysis, Data curation, Conceptualization.

## Declaration of competing interest

The authors declare the following financial interests/personal relationships which may be considered as potential competing interests: Garima Sharma reports financial support was provided by American Heart Association Inc. If there are other authors, they declare that they have no known competing financial interests or personal relationships that could have appeared to influence the work reported in this paper.
